# Temperature and CO_2_ alter trophic structure of Arctic plankton assemblages

**DOI:** 10.1038/s41598-025-10591-0

**Published:** 2025-08-20

**Authors:** Koji Sugie, Bingzhang Chen, Shigeto Nishino, Toru Hirawake

**Affiliations:** 1 Research Institute for Global Change, Japan Agency for Marine-Earth Science and Technology (JAMSTEC), 2-5, Natsushima-cho, Yokosuka, 237-0061 Kanagawa Japan; 2 Department of Mathematics and Statistics, University of Strathclyde, Glasgow, UK; 3 Faculty of Fisheries Sciences, Hokkaido University, 3-1-1 Minato-cho, Hakodate, Japan; 4 Present address, National Institute of Polar Research/SOKENDAI, 10-3, Midori-cho, Tachikawa, Tokyo, 190-8518 Japan

**Keywords:** Climate change, Ocean acidification, Phytoplankton, Microzooplankton, Ecology, Biooceanography, Climate-change ecology, Marine biology

## Abstract

Driven by increasing anthropogenic CO_2_, the impact of ongoing climate change on the marine plankton ecosystem ultimately extends to higher trophic levels and the biogeochemical cycling of carbon and nutrients. However, the impacts of multiple environmental changes on trophic interactions between predator and prey have still not been fully explored. Here we conducted incubation experiments to determine the temperature and CO_2_ sensitivities of marine phytoplankton growth and microzooplankton grazing in the western Arctic Ocean, where rapid climate change is taking place. The temperature sensitivity of the growth of larger phytoplankton decreased owing to the increase in CO_2_ levels, whereas that of the growth of smaller phytoplankton increased under higher CO_2_ levels. Notably, the temperature sensitivity of Arctic phytoplankton is at least two times higher than the canonical estimates irrespective of size classes, highlighting the uniqueness of the Arctic ecosystem’s response to warming. Microzooplankton grazing was closely coupled with, but did not exceed, the growth rates of their prey, suggesting that microzooplankton behavior is mainly regulated by prey availability rather than the ambient environment. The higher competitiveness of smaller phytoplankton under higher temperatures and CO_2_ conditions might lead to a less productive Arctic Ocean ecosystem for higher trophic-level organisms in the future.

**Supplementary Information:**

The online version contains supplementary material available at 10.1038/s41598-025-10591-0.

## Introduction

**Fig. 1:**
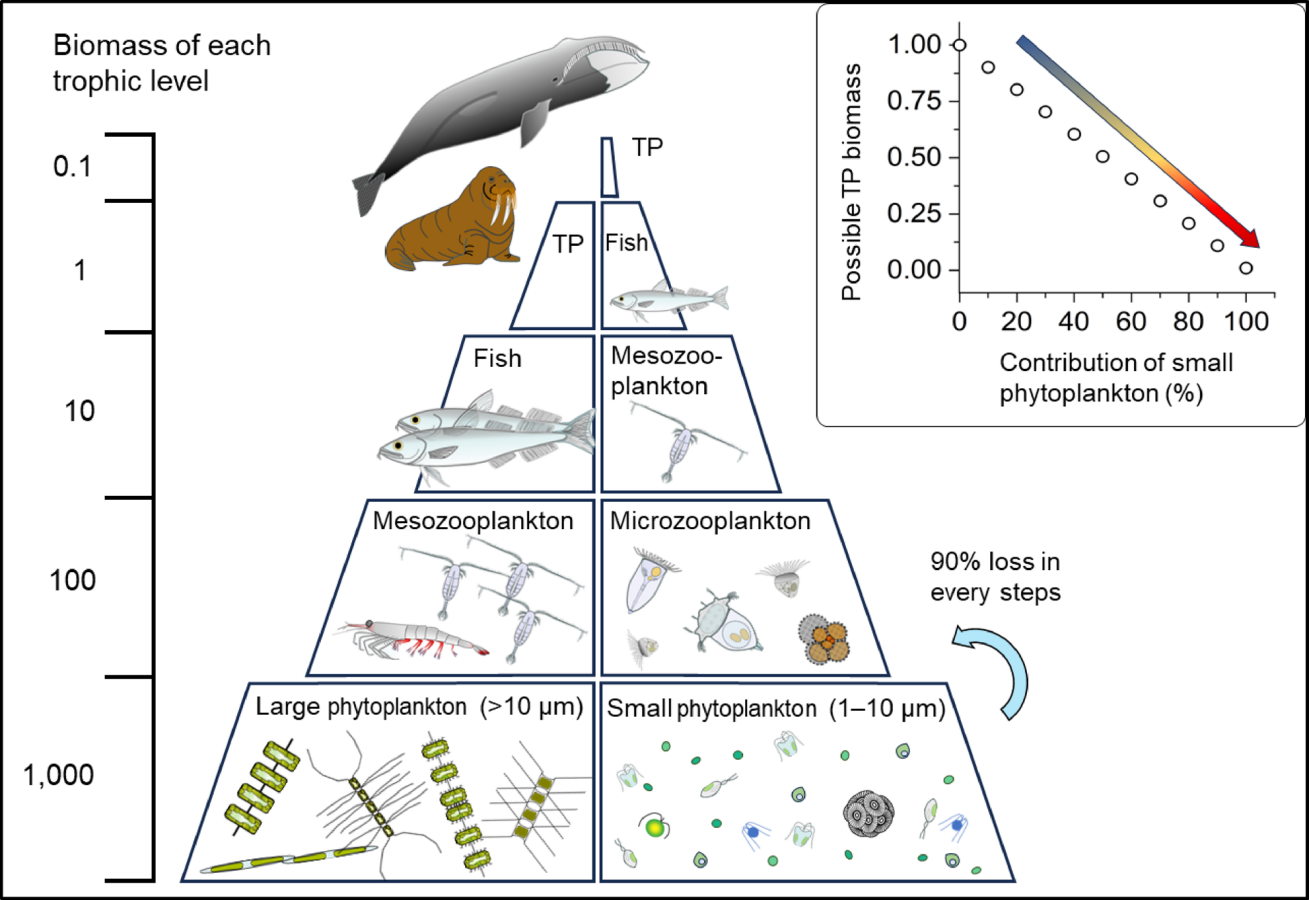
Simplified two ecological pyramids basing (left) large and (right) small phytoplankton assemblage. Given each ecosystem started the same primary production (1000), the production of fish and top predators (TP) can dramatically change because of the difference in the size of phytoplankton or the number of lower trophic levels (upper right panel). A constant trophic transfer efficiency at 10% is applied here.

Ocean warming and acidification, caused by an increase in anthropogenic carbon dioxide (CO_2_)^1,2^ and temperature rise in recent decades, shift the biogeography of planktonic organisms poleward^3,4^. The pace of warming in the Arctic Ocean is nearly four times that measured in the lower latitudes^5^and this warming trend is expected to continue^6,7^. However, organisms that are already in the polar region are unable to shift further poleward and must cope with future climate change. Phytoplankton, which form the base of the marine ecosystem (Fig. 1), are responsible for nearly half of the global primary production^8^ so these changes could extend to the production of higher trophic levels and alter the biogeochemical cycling of bio-elements.

Changes in phytoplankton biogeography, productivity, and phenology owing to environmental changes have already appeared in the Arctic Ocean^7^. Because temperature is a prime factor for influencing metabolic rates^9–11^ and CO_2_ is the sole substrate for photosynthesis of photolithoautotrophs^12,13^ anthropogenic CO_2_-induced climate change could modify all aspects of phytoplankton behavior in ecosystem processes. Previous studies suggest that smaller, pico-sized phytoplankton (< 2 μm), which are inefficient components in the production of higher trophic levels (Fig. 1), would be more dominant under the expected future high temperature and CO_2_ levels of the Arctic Ocean^14–16^. However, it is unclear whether the increase in smaller phytoplankton abundance is a result of the faster growth rate of smaller phytoplankton or the decline of grazing pressure under temperature and/or CO_2_ perturbations. Most previous studies investigated the effect of each environmental variable on a single species and single group of interest (e.g., monoculture, phytoplankton, copepod, calcifying algae or mollusks)^1,10,17,18^. However, the impacts of multiple environmental perturbations on marine organisms across different trophic levels remain unclarified to date^1,19^. Here, we conducted temperature and CO_2_ perturbation experiments using Arctic plankton communities in 2017 and 2018 (Supplemental Fig. 1) to assess the sensitivities of phytoplankton growth and microzooplankton grazing to climate change.

## Materials & methods

To measure both phytoplankton growth and microzooplankton grazing rates under different temperatures and CO_2_ levels, we conducted four sets of two-point dilution technique incubation (Supplemental Fig. 2)^20,21^ under the combinations of in situ temperature and CO_2_ levels (control: LT treatment), CO_2_-added to the controls (LT plus high CO_2_: LTHC), 4 °C higher temperature relative to the controls (HT), and CO_2_-added to the HT treatment (HTHC). Our selected temperature increase of 4 °C represents the global average warming between the preindustrial era and the end of this century under the RCP8.5 scenario, which is in line with the expected future Arctic under the RCP4.5 scenario^22^. Approximately 100–200 µmol kg^−1^ of dissolved inorganic carbon was added to the HC series (798 ± 298 µatm, *n* = 18; Supplemental Table 1) to achieve the expected high CO_2_ levels estimated at the end of this century under the RCP8.5 scenario^2^.

Seawater samples for the two-point dilution experiments were collected from the northern Bering Sea and the Chukchi Sea of the western Arctic Ocean aboard the *R/V* Miari (Japan Agency for Marine-Earth Science and Technology) from August to September 2017 and the *T/S* Oshoro-Maru (Hokkaido University) in July 2018 (Supplemental Fig. 1; Supplemental Table 1). Seawater was collected at a depth of 10 m using acid-washed, Teflon-coated 12-L Niskin-X sampling bottles (General Oceanics, Miami, FL, USA) attached to a conductivity, temperature, and depth profiler with a carousel water sampler (CTD-CWS; Sea-Bird Scientific, Bellevue, WA, USA). For undiluted seawater samples, seawater was sieved through an acid-washed 200 μm mesh attached to silicon tubes to remove large plankton, and the outlet of the seawater was placed on the bottom of a 9-L polycarbonate (PC) tank to reduce physical damage to protists (Supplemental Fig. 2). For filtered seawater (FSW), a 0.2 μm pore-size capsule cartridge filter (Pall Corp., Washington, NY, USA) was used and poured into a 9-L PC tank and a 100-mL polypropylene bottle. Nutrients were added to two 9-L polycarbonate bottles to make final concentrations of nitrate, ammonia, phosphate, and silicic acid of 10, 5, 1, and 15 µmol L^−1^, respectively, and gently homogenized. Then, 200 μm sieved and filtered seawater samples were gently dispensed into two sets of four 2-L PC bottles, respectively. For the HC treatment, the FSW collected in a 100-mL polypropylene bottle was bubbled using 100% CO_2_ gas for 0.5 to 1 h to make 1 atm *p*CO_2_ FSW, and about 4 to 5-mL of high CO_2_ FSW was spiked into four 2-L PC bottles: two for FSW and two for 200 μm mesh sieved seawater. For the different temperatures, two sets of four 2-L PC bottles (two FSW and two 200 μm mesh sieved seawater) were then incubated in two on-deck water tanks for 24 h, which were set at in situ temperature and the in situ temperature plus ca. 4 °C. The temperature in the water tanks was controlled using thermostatic circulators (RX-602AN, IWAKI Co. Ltd., Tokyo, Japan) and monitored using thermistor sensors (DEFI-T, JFE Advantech Co. Ltd., Hyogo, Japan). Twenty-four hours acclimation periods were established to acclimate the plankton assemblages to different light, temperature, and CO_2_ levels to avoid unintended and unpredictable growth lag and chlorophyll-*a* bleaching^23,24^. After 24 h of acclimation, the following procedures were conducted for each treatment to start the dilution experiment^21^; the 200 μm sieved seawater was gently dispensed into two 600 mL PC bottles and another two (2017 experiment) or three (2018 experiment) 600-mL PC bottles were filled with 70-mL of 200 μm sieved seawater and 530 mL of the FSW (88% dilution). The remaining 200 μm sieved seawater was used for the samples at the beginning of the experiment. The duplicate non-diluted 200 μm sieved seawater and duplicate (2017 experiment) or triplicate (2018 experiment) 88% diluted seawater with FSW were incubated for 24 hours^20,21,24^. In 2017, one bottle of 88% diluted seawater sample was sacrificed for the samples at the beginning of the dilution experiment. However, the difference in the results between the direct measurement of 88% diluted water and the multiplied dilution factor (12%) of non-diluted 200 μm sieved seawater was less than a few percent (data not shown). Therefore, the 88% diluted seawater samples were incubated for dilution experiments in 2018 to enhance statistical robustness.

During sampling, at the start (after 24 h of acclimation) and the end of the 24 h of incubation, samples for size-fractionated chlorophyll-*a* (chl-*a*) were collected by filtering 580-mL (12% diluted treatments) or 200 to 300-mL (non-diluted treatments) of seawater sequentially using a 10 μm pore-size PC membrane and a GF/F (nominal pore size of 0.7 μm) filters under a gentle vacuum. Filter samples were soaked in *N*,* N*-dimethylformamide for at least 24 h at − 20 °C in the dark. The extracted chl-*a* was measured with fluorometry using a Turner Designs 10-AU fluorometer^25^. Samples for the enumeration of small phytoplankton such as pico-cyanobacteria, pico-eukaryotes (< 2 μm), and nano-eukaryotes (2–10 μm) were fixed using glutaraldehyde and stored in a deep freezer at − 80 °C until on-land laboratory analysis, which was performed using a flow cytometer (EC800 Flowcytometry Analyzer, SONY Corp., Tokyo, Japan). Plankton measurement more than approximately ~ 10 μm were examined by inverted microscope under 100×, 200×, or 400× magnifications (Olympus Corp., Tokyo, Japan). Through these microscopic observations, the biomass of diatoms and microzooplankton (mainly composed of heterotrophic dinoflagellates, ciliates, rotifers, and copepod nauplii) was determined by measuring the size of the organisms and converting the data using allometry^26,27^. Analyses of nutrients (NO_3_, NO_2_, NH_4_, PO_4_, and Si(OH)_4_), dissolved inorganic carbon, and total alkalinity were done as described in our previous study^16^. The chemical variables were measured using the appropriate certified reference materials (Supplemental Tables 1–3)^28–30^.

The apparent growth rate of phytoplankton in each treatment was estimated using the difference of the natural log chl-*a* concentration between the start and the end of 24 h of incubation (µ_app_). The intrinsic phytoplankton growth rate (µ) was estimated from the y-intercept of a regression of µ_app_ as a function of the FSW dilution factor (0% and 88% in this study), representing the phytoplankton growth rate with no microzooplankton grazing. The slope of the above regression, or µ minus µ_app_ in the non-diluted 200 μm sieved seawater represents the grazing mortality rate by microzooplankton^20,21^. One of the major problems in estimating µ using chl-*a* concentrations is the change in the cellular chl-*a* level in response to the change in light conditions between the in situ and incubation conditions^24^. Additionally, approximately 1 day of phytoplankton growth lags in response to temperature and CO_2_ manipulations were observed in our previous study^16^. To minimize the above phenotypic plasticity of cellular chl-*a* levels, we implemented the 24 h acclimation period for the plankton community under each incubation condition.

Temperature and CO_2_ sensitivity indices were estimated from the following equation: Temperature sensitivity = (*µ*_HT_ − *µ*_LT_)/(*T*_HT_ − *T*_LT_) or (*µ*_HTHC_ − *µ*_LTHC_)/(*T*_HTHC_ − *T*_LTHC_), and high CO_2_ sensitivity = (*µ*_LTHC_ − *µ*_LT_)/(*p*CO_2LTHC_ − *p*CO_2LT_) × 100 or (*µ*_HTHC_ − *µ*_HT_)/(*p*CO_2HTHC_ − *p*CO_2HT_) × 100, respectively. We replaced µ with m when estimating the indices of grazing mortality. We multiplied the high CO_2_ sensitivity index by 100 to make it comparable to the temperature sensitivity index based on the expected atmospheric CO_2_ increase from 280 to approximately 700 ppm and the resulting temperature rise of approximately 4 °C from preindustrial time to the end of this century in the RCP8.5 scenario, i.e., ~ 105 ppm CO_2_/°C. We also applied temperature sensitivity in exponential equation using the Eppley curve with µ = a*e*^bt^, where a, b, and t represent an intercept of regression, temperature coefficient, and temperature (°C), respectively. We could not estimate activation energy using the Arrhenius-van’t Hoff equation in metabolic theory of ecology^11,31^ because it requires data more than three temperature treatments.

We analyzed the mean values of the measured specific growth rates among the different treatments with the two-way ANOVA and post-hoc multiple comparison with the Holm-Bonferroni method to check the interactive effects of temperature and CO_2_ levels or temperature/CO_2_ and different sizes of protists on the growth and grazing rates of marine protists (Supplemental Tables 4–6). Because the interactive effect was detected only in a few cases with relatively lower probability, we omitted it from the analyses in this study. The statistical analyses were performed using Origin software (ver. Origin Pro 2020; Lightstone Corp., Tokyo, Japan; https://www.lightstone.co.jp/origin/) and reported at a 95% confidence level. The results are shown as the mean ± 1 SD of duplicate (2017 experiments) or triplicate incubations (2018 experiments).

## Results & discussion

**Fig. 2:**
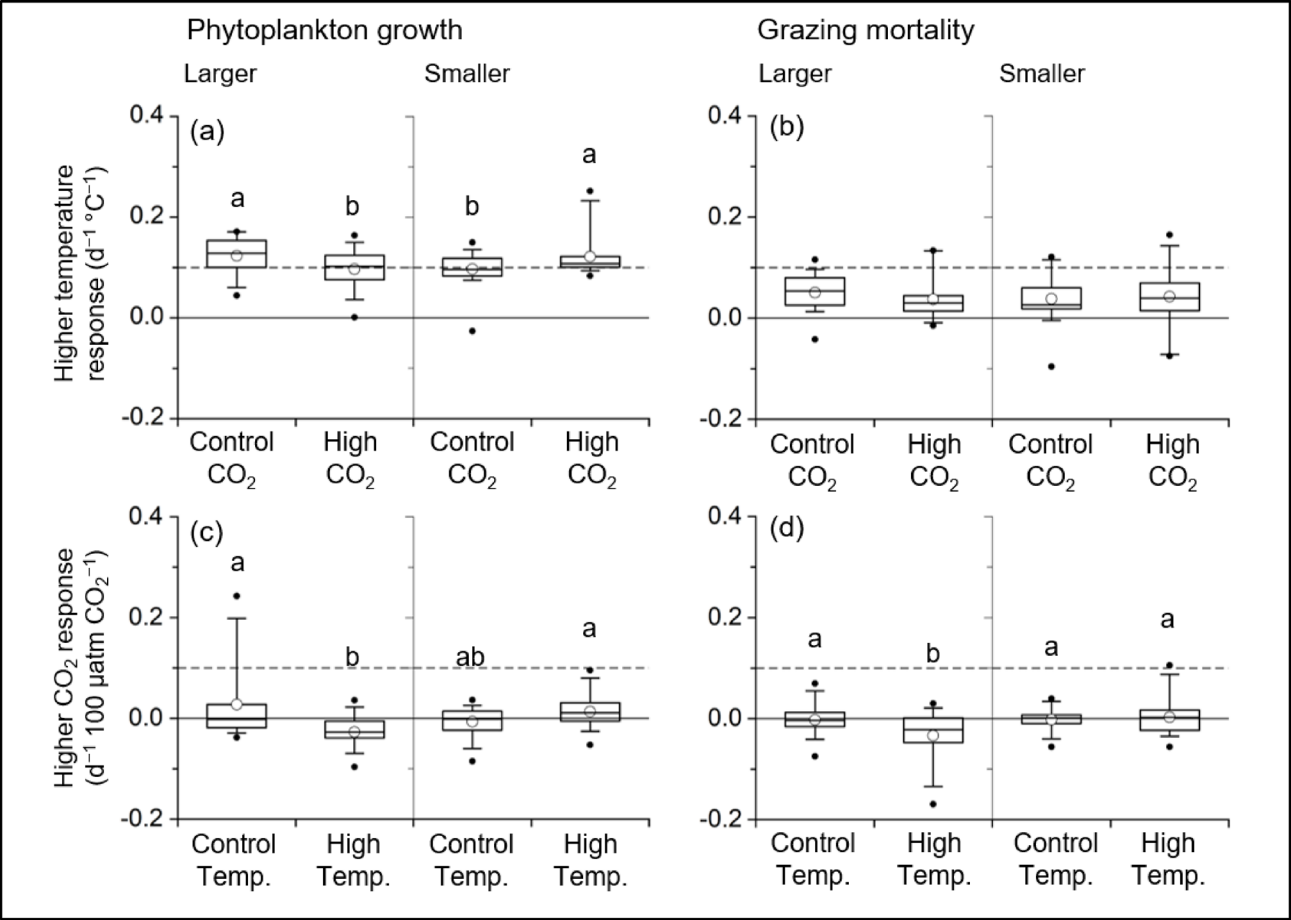
Temperature (T) response of (**a**) the specific growth rates (µ) of larger (> 10 μm) and smaller (< 10–0.7 μm) phytoplankton and (**b**) grazing mortality rate (m) on larger and smaller phytoplankton. High CO_2_ response of (**c**) the specific growth rates of larger (> 10 μm) and smaller (< 10–0.7 μm) phytoplankton and (**d**) grazing mortality rate on larger and smaller phytoplankton. Letters above the box diagram represent statistical results of two-way ANOVA and post-hoc multiple comparisons with the Holm-Bonferroni method.

Higher temperature enhanced the growth of both larger (> 10 μm) and smaller (< 10–0.7 μm) phytoplankton groups at mean rates of 0.123 and 0.097 d^−1^ °C^−1^, respectively, under control CO_2_ conditions (Fig. 2a; Supplemental Figs. 3–6). Under higher CO_2_ conditions, temperature sensitivity was significantly reduced by 22% for larger phytoplankton, whereas it increased by 25% for smaller phytoplankton when compared with those at the control CO_2_ levels (Fig. 2a). The temperature response of grazing mortality rates showed a similar pattern to that of the phytoplankton growth rates, but these differences were statistically insignificant (Fig. 2b; Supplemental Figs. 3–6). The sensitivity of the growth rates of larger phytoplankton to higher CO_2_ levels significantly decreased under higher temperature conditions, whereas it significantly increased for smaller-sized phytoplankton (Fig. 2c). The higher CO_2_ response index in grazing mortality rate also showed a similar pattern to that observed in phytoplankton growth. Namely, the index significantly decreased in the grazing rate on larger phytoplankton under higher temperature (Fig. 2d). These results suggest that the predominance of smaller phytoplankton at higher temperature and CO_2_ conditions seen in previous studies^15,16,18,32^ may result from the higher temperature and CO_2_ sensitivities of their growth and tight coupling but incomplete grazing pressure of microzooplankton.

A possible explanation for the higher fitness of smaller phytoplankton in the Arctic Ocean under higher temperatures and CO_2_ levels is that the form of the inorganic carbon in their photosynthesis is largely dependent on aqueous CO_2_, rather than HCO_3_^16,18,32^. Because aqueous CO_2_ concentration increases thermodynamically with temperature, both higher temperature and CO_2_ levels enhance aqueous CO_2_ concentrations. Additionally, the *p*CO_2_ levels in this study were often less than 300 µatm (Supplemental Table 3) in the Arctic Ocean during summer because of active CO_2_ uptake by phytoplankton prior to the sample collections, which were performed during late spring to summer^7,33^. Those conditions could have led to the higher fitness of smaller phytoplankton in response to the higher temperature and CO_2_ levels (Fig. 2). In contrast, larger phytoplankton groups, which most likely consisted of diatoms in the study area, use both aqueous CO_2_ and HCO_3_^−^ for photosynthesis which can modify the outcome of temperature and CO_2_ responses, especially under low CO_2_ conditions.

Under low *in situ p*CO_2_ levels, we found a large deviation in the CO_2_ response index of large-sized phytoplankton for the control and higher temperature conditions (Supplemental Fig. 7). The positive impact of CO_2_ addition in the control temperature condition decays exponentially with increasing *in situ p*CO_2_ levels, whereas the negative impact of CO_2_ addition appeared in the higher temperature conditions with decreasing *in situ p*CO_2_ levels (Supplemental Fig. 7). Under low ambient CO_2_ conditions, large-sized phytoplankton such as diatoms need to upregulate their carbon concentration mechanisms, including intracellular and extracellular carbonic anhydrase to concentrate CO_2_ around Rubisco, the enzyme that catalyzes CO_2_ into organic matter^12,13^. Additionally, the Rubisco abundance of marine phytoplankton, which includes large-sized diatoms, increased under lower CO_2_ levels^12,34,35^ indicating that lower CO_2_ levels potentially impose a heavy cost on the dark reaction (Calvin cycle) of photosynthesis. Therefore, the addition of CO_2_ should have improved the growth of photoautotrophs under lower CO_2_ levels as observed in the present study under the control temperature conditions (Supplemental Fig. 7). In contrast, under the higher temperature condition, the additional costs of temperature-dependent respiration^10,36^ and enhancing HCO_3_^−^ uptake using chloroplast pumping, which requires respirative energy^37^ may lead to the delayed the growth of larger phytoplankton in response to CO_2_ addition.

**Fig. 3:**
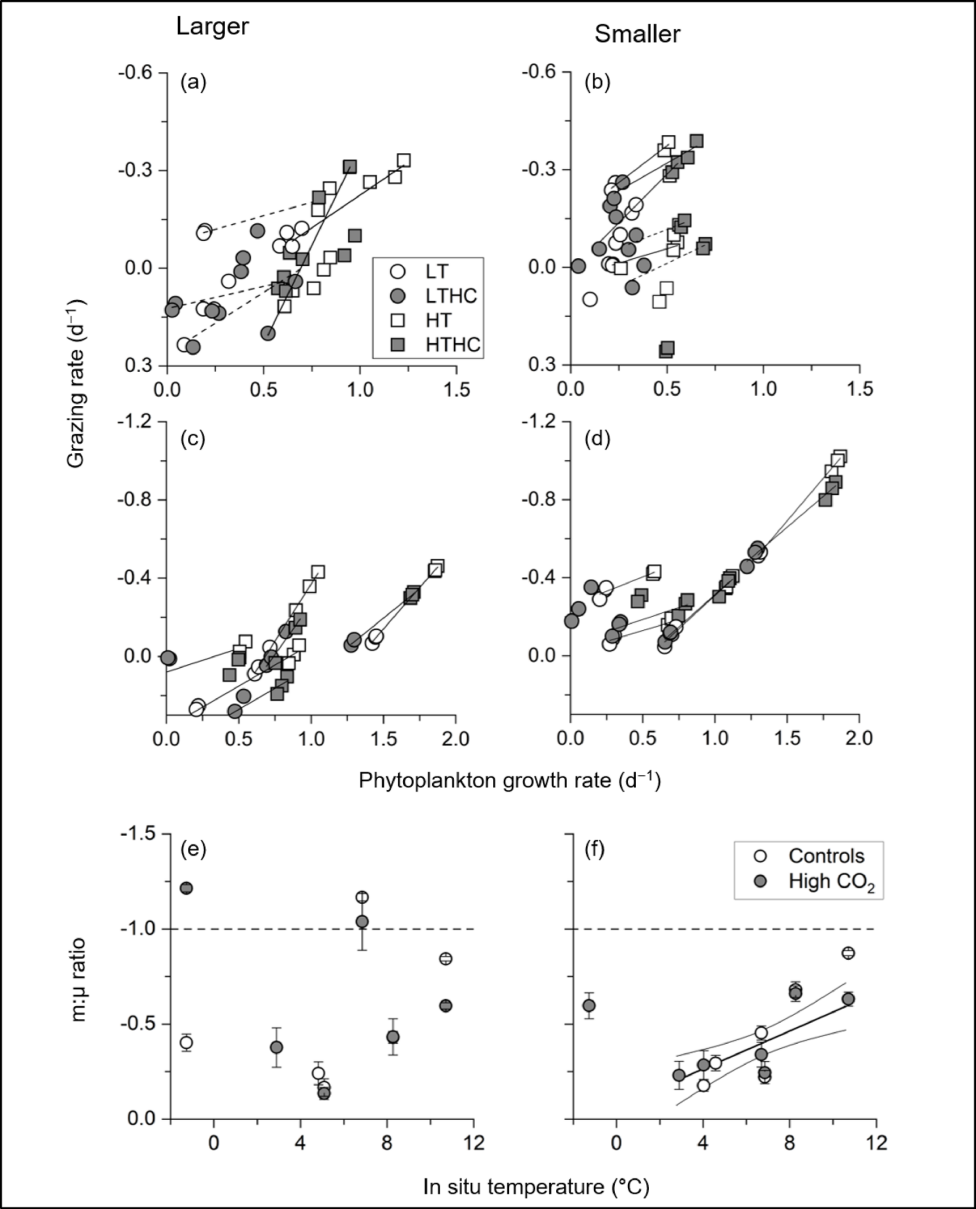
Relationships between phytoplankton growth rate (µ) and grazing mortality rate (m) of (**a**), (**c**) larger and (**b**), (**d**) smaller traits in (**a**), (**b**) 2017 and (**c**), (**d**) 2018 experiments. Solid (*p* < 0.01) and dashed lines (*p* < 0.05) represent significant correlations between different temperature treatments (LT and HT or LTHC and HTHC). The slope of the above correlation represents the m:µ ratio under either control CO_2_ or high CO_2_ conditions. The relationship between the m:µ ratio and in situ temperature where samples were collected at each station, for (**e**) larger and (**f**) smaller traits.

Most previous studies on natural plankton communities with temperature and/or CO_2_ manipulated incubation were conducted in non-diluted seawater samples with a mixture of phytoplankton and zooplankton. This means that the observed results, such as the higher competitiveness of smaller phytoplankton under high temperature/CO_2_ levels^15,16,18^ should be the consequences of the apparent phytoplankton abundance, which had experienced extensive zooplankton grazing^20,38–40^. In this study, phytoplankton growth rate and grazing mortality rate were generally coupled, but the grazing mortality rate, temperature, and CO_2_ sensitivities of microzooplankton rarely exceeded those of phytoplankton (Figs. 2 and 3; Supplemental Figs. 8 and 9), indicating that the smaller phytoplankton can accumulate biomass under nutrient- and light-replete conditions, even in the higher temperature treatment in the Arctic Ocean. Our results clearly indicate that examining the impacts of environmental changes on phytoplankton assemblage using non-diluted seawater partially masks these environmental perturbations due to microzooplankton grazing on smaller traits, or alternatively, the impacts become more apparent for larger traits^15,18,35^.

We then estimated the relationship between phytoplankton growth (µ) under the LT and HT or between the LTHC and HTHC treatments and grazing mortality rates (m) in each incubation experiment. The slope represents the m:µ ratio (Fig. 3, Supplemental Figs. 8 and 9) to characterize a temperature-dependent trophic interaction. The m:µ ratio in the large-sized traits showed no significant relationship with the in situ seawater temperature, whereas that in the small-sized traits had a significant positive correlation with temperature when excluding the data for the ice-edge assemblage at St. 89 in 2017 at negative temperatures (Fig. 3e and f; Supplemental Fig. 1). Previous studies suggested unique trophic interactions under near-freezing temperatures of the Arctic Ocean, such that the growth and grazing rates of microzooplankton were sporadically high enough to exhaust primary production of phytoplankton, i.e., the m:µ ≥ 1^refs. 39, 40^. According to the laboratory mono-species experiments and dilution experiments using natural plankton communities, the temperature sensitivity of microzooplankton growth is potentially higher than that of phytoplankton^40–42^. Although determining the temperature sensitivities of the ice-edge assemblages in the Arctic Ocean require more data, our results partially support the above hypothesis that the microzooplankton grazing rate approaches the phytoplankton growth rate with increasing seawater temperature in the smaller-sized community. This suggests that any further increase in temperature could enhance nutrient recycling ecosystems between smaller phytoplankton and microzooplankton which is potentially lower productivity of higher trophic levels (Fig. 1).

**Fig. 4:**
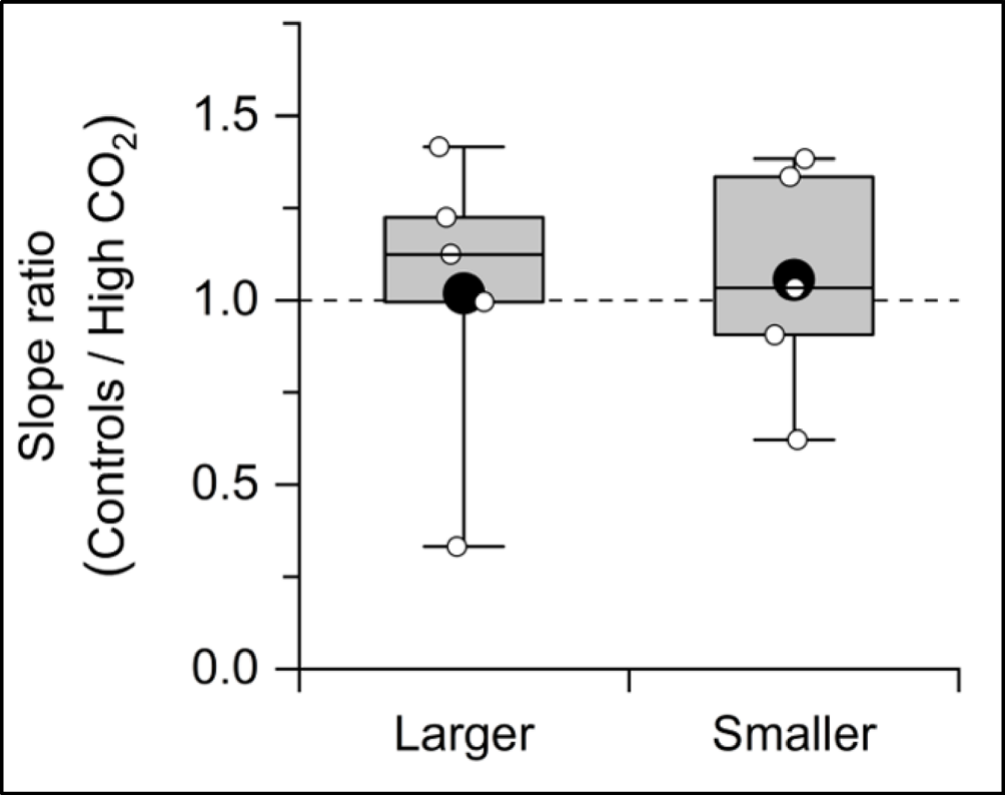
Box diagram of the m:µ slope ratio between the control CO_2_ and higher CO_2_ levels in each dilution experiment. The black circles, boxes, and horizontal bars within a box and bars represent the mean values of m:µ, ranges for 25–75% of data, median values of m:µ, and ranges for 1–99% of data, respectively. Small white circles represent estimated slope ratios based on the result of this study.

In most previous studies^43–45^ the impact of environmental changes on microzooplankton assemblages could not distinguish between direct effects and indirect effect mediated via phytoplankton responses, due to the tight coupling between phytoplankton growth and microzooplankton grazing (Fig. 3). To assess the sensitivity of microzooplankton activity to higher CO_2_ levels, we compared the m:µ ratio (m normalized by µ) between the control and higher CO_2_ levels (m:µ_controls_/m:µ_high−CO2_). The CO_2_ sensitivities in both size classes were not significantly different from the unity, indicating that the CO_2_ levels did not directly affect microzooplankton (Fig. 4). Although previous studies did not consider phytoplankton behavior in the microzooplankton response to environmental perturbations, those studies also suggested that microzooplankton grazing rates and species composition were not affected by high CO_2_ conditions^43–45^. Using the phytoplankton growth-normalized grazing rate (i.e., m:µ ratio) under different CO_2_ levels, we provide more robust evidence that higher CO_2_ levels did not directly affect microzooplankton grazing activity but indirectly via phytoplankton growth rate in the Arctic Ocean. We suggest that estimating microzooplankton grazing activity should be a temperature-dependent change proportional to the growth rate of smaller phytoplankton traits under nutrient-replete conditions (Fig. 3). In contrast, predictions of grazing pressure on larger phytoplankton traits were difficult from our results, likely because of the heterogenous presence of small copepods and phagotrophic/pallium-feeding dinoflagellates in the incubation bottle or the presence/absence of large colony-forming algae^40,46^.

**Fig. 5:**
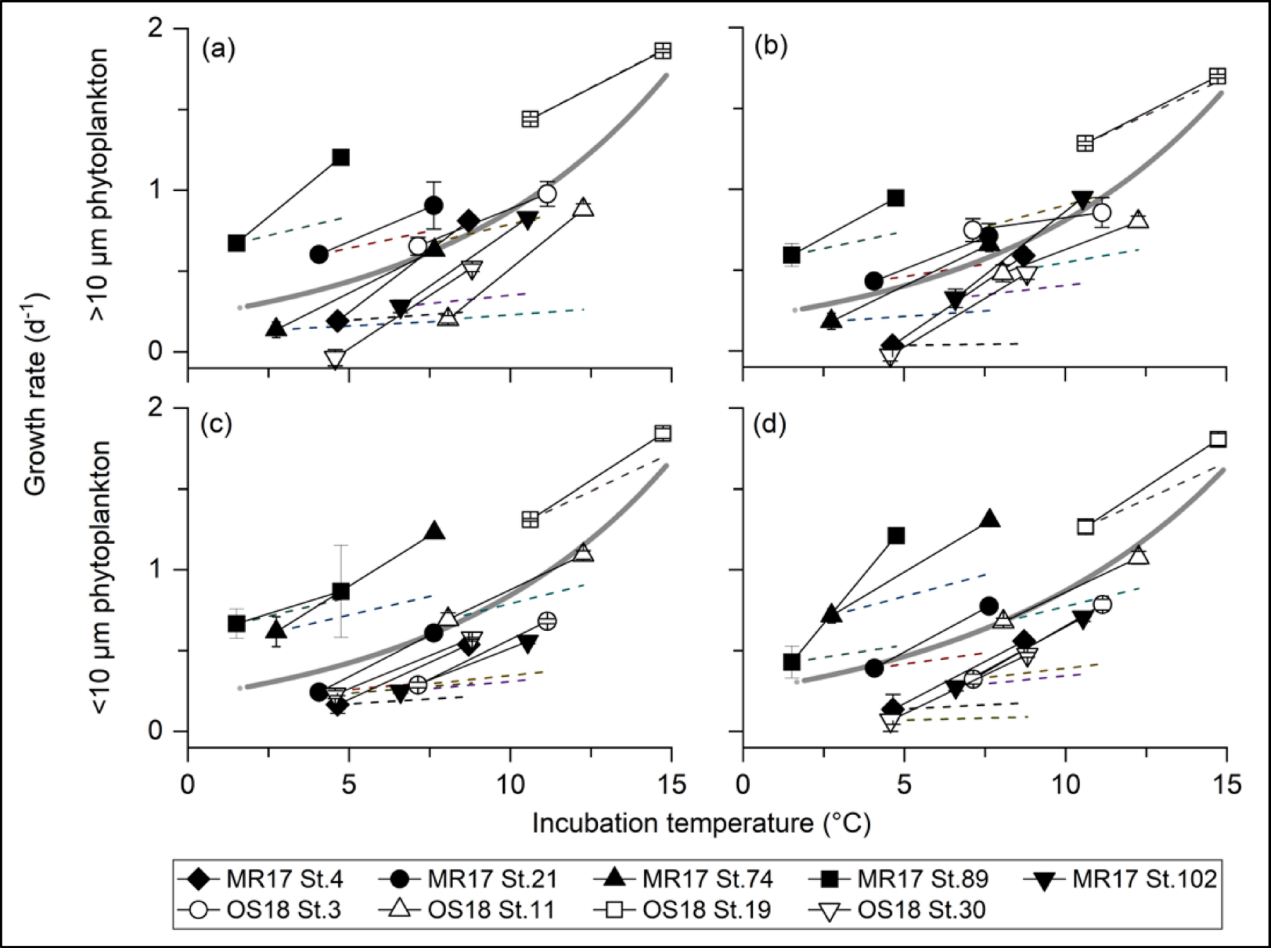
Relationship between incubation temperature and the growth rates (µ) of (**a**, **b**) > 10 μm and (**c**, **d**) < 10 μm phytoplankton traits. (**a**, **c**) and (b, **d**) represent the controls and higher CO_2_ levels, respectively. Thin lines represent the temperature response between LT and HT or LTHC and HTHC treatments observed in this study. Dashed lines represent the estimated temperature response of phytoplankton using the canonical value of Q_10_ = 1.88 (Eppley 1972). Gray bold lines represent exponential fitting with µ = a*e*^bt^ (see method). (**a**) µ = 0.22*e*^0.14t^ (*p* < 0.001, *F*_2,16_ = 46.3, R^2^ = 0.48), (**b**) µ = 0.20*e*^0.14t^ (*p* < 0.001, *F*_2,16_ = 63.9, R^2^ = 0.58), (**c**) µ = 0.21*e*^0.14t^ (*p* < 0.001, *F*_2,16_ = 52.3, R^2^ = 0.49), and (**d**) µ = 0.25*e*^0.13t^ (*p* < 0.001, *F*_2,16_ = 45.3, R^2^ = 0.42).

The observed phytoplankton temperature sensitivity of approximately 0.1 d^−1^ °C^−1^ is much higher than the canonical estimates, especially for the incubations under low temperatures (Fig. 5). The phytoplankton growth rate is generally expressed as a function of temperature in the exponential curve (µ = a*e*^bT^, a: intercept at temperature T = 0, b: temperature coefficient) using Q_10_ (Q_10_ = (µ_T2_/µ_T1_)^(10/(T2−T1)^, where µ_T1_ and µ_T2_ represent phytoplankton growth rate at seawater temperature T1 and T2 (T1 < T2), respectively) or activation energy theories^9,11,47–49^. Therefore, the expected change in the growth rate under low temperatures (< 10 °C) with slow intrinsic phytoplankton growth rate in the exponential equation is on average 8.8- and 4.7 times smaller than the measured change in growth rates of larger and smaller traits, respectively (Fig. 5)^9,31,50^. Given that temperature sensitivity is linear at a rate ranging from ~ 0.1 to 0.13 d^−1^ °C^−1^ as estimated in the present study (Fig. 2a), the estimated Q_10_ value easily exceeds more than 10 with decreased growth rate of phytoplankton in the polar region (Supplemental Fig. 10). Previous observation and modeling studies modified the intercept of the curve to be higher, ranging from 0.59 to 0.81^refs. 9, 31, 50^, or use a higher temperature coefficient (b > 0.063 or Q_10_ > 1.88) to represent polar ecosystem dynamics^51,52^. The empirical exponential relationship in our study showed a lower intercept (0.2–0.25) and temperature coefficient that was two times higher (b ≈ 0.14 or Q_10_ ≈ 4; Fig. 5) than that of the canonical estimates, indicating that the previous studies potentially overestimate the intrinsic growth rates of phytoplankton under lower temperature conditions and underestimate the temperature sensitivity of polar phytoplankton. Our results strongly support the suggestions of previous studies^39,40,51,52^ in that the temperature-related change in the productivity of lower trophic levels in the Arctic Ocean is much more dynamic than the estimates based on the canonical exponential curve.

The empirical exponential curve of this study still underestimates the temperature sensitivity of each incubation of low-temperature assemblages and vice versa under higher temperatures (Fig. 5). Theoretical considerations using laboratory monoculture data suggested that the temperature sensitivity of each species is higher than the empirical relationship of the pooled dataset^11,48^. A similar trend has been observed using the phytoplankton communities in the subtropical North Pacific in that the temperature sensitivity of each community was higher than that of the metapopulation in the region^49^. The higher temperature sensitivities of individual species and each natural assemblage^11,49^ indicates that the Eppley curve may be inappropriate function to represent the growth rates with the same phytoplankton assemblage under the seasonal temperature gradient but suitable to represent the maximum growth rate of global ocean. Because the polar region has a geographical limitation to poleward shift in biogeography with climate warming^3,4^ the temperature sensitivity of each local community may be important for better understanding the present seasonality and future ecosystem change, rather than extrapolating that of global metapopulation. However, the possibility of the range shift of subarctic and temperate plankton to polar regions in the face of climate change requires careful attention because it may alter species composition and, potentially, the temperature and CO_2_ sensitivities of lower trophic levels^4,15,16,53,54^.

The lower temperature sensitivity expected at the lower end of exponential models, such as the Eppley curve, likely results from limitations in the model equation, which is based on a relatively small number of data points from unialgal culture exhibiting similar maximum growth rates^9,31,50^. The temperature sensitivities of seawater chemistry and biochemistry are expected to exhibit relatively consistent rates across a range of temperatures. Accordingly, it is reasonable to assume that individual organisms, as integrated systems of these chemical and biochemical processes, may also display relatively constant temperature sensitivities. This assumption is supported by our observations, which showed a growth rate response of approximately 0.10 d⁻¹ °C⁻¹ (Fig. 2). However, further investigation is needed to confirm this hypothesized chemical, biochemical, and physiological consistency. Notably, our results indicate that CO₂ can modulate this otherwise stable temperature sensitivity because of the differences in inorganic carbon utilization strategies among phytoplankton species^12–14,32,37^. To our knowledge, no previous studies have simultaneously examined the interactive effects of temperature and CO₂ on the different size class of plankton communities using dilution technique. Nevertheless, it is plausible that similar synergistic environmental changes in other marine systems could significantly alter the size structure and dynamics of planktonic communities.

This study shows that the combined effects of higher temperature and CO_2_ levels in the Arctic Ocean can shift the size composition of phytoplankton to be smaller and the temperature-dependent m:µ coupling to be tight, resulting in an efficient nutrient-recycling ecosystem. Because smaller phytoplankton are generally winners in the nutrient-recycling ecosystems^19,20^ the combination of higher temperature and CO_2_ levels act as negative feedback to the current larger-phytoplankton dominated, productive Arctic ecosystem, which includes large mammals (Fig. 1)^7,55^. The nutrient levels in the western Arctic Ocean in late summer to autumn are generally low enough to regulate phytoplankton growth rates^7,56^ but this study was conducted in nutrient-replete conditions. Our study may contribute to a better understanding of the ecological response to spring phytoplankton blooms and sporadic events of nutrient pulse during summer to autumn^57,58^. In other words, the simple installation of temperature and CO_2_ sensitivities estimated from our results to numerical simulations requires caution because nutrient limitation can regulate the temperature sensitivity of phytoplankton metabolisms^59^. Whilst this study has limitations in spatial and temporal resolutions, the higher temperature sensitivity of phytoplankton assemblages should be an important mechanism for supporting the productivity of higher trophic levels, including polar bears and seabirds^55^. However, because further increases in CO_2_ levels can negatively affect the production of top predators by altering size composition of phytoplankton, the reduction of anthropogenic CO_2_ emission is an urgent issue for sustaining the health of Arctic ecosystem.

## Electronic supplementary material

Below is the link to the electronic supplementary material.

Supplementary Material 1

Supplementary Material 2

Supplementary Material 3

Supplementary Material 4

Supplementary Material 5

Supplementary Material 6

## Data Availability

The datasets used and/or analyzed during the current study available from the corresponding author on reasonable request.
