# Supplementary material for: Temperature and CO2 alter trophic structure of Arctic plankton assemblages
Source: Sci Rep. 2025 Aug 20;15:28582. doi: 10.1038/s41598-025-10591-0 (PMC12365226; doi:10.1038/s41598-025-10591-0)
Supplement: Supplementary file 1 — Supplementary Material 1 [file 41598_2025_10591_MOESM1_ESM.docx]

Supplemental Table 3. Environmental variables and carbonate chemistry during the manipulation experiments.

Cruise Stn. No. Treat. Temp. DIC TA pH *p*CO_2_ PAR

(°C) (µmol kg^−1^) (µmol kg^−1^) (µatm) (mol m^−2^ d^−1^)

MR17 St. 4 LT 4.65 2050.0 2221.1 8.130 312.3 8.76

LTHC 4.65 2225.2 2220.6 7.584 1218.4 8.76

HT 8.71 2046.5 2223.9 8.083 358.3 8.76

HTCH 8.71 2196.8 2221.4 7.631 1117.2 8.76

MR17 St. 21 LT 4.07 1977.6 2159.8 8.192 259.8 4.41

LTHC 4.07 2146.8 2159.8 7.667 973.4 4.41

HT 7.63 1978.6 2161.2 8.137 303.3 4.41

HTCH 7.63 2140.2 2158.9 7.638 1067.8 4.41

MR17 St. 74 LT 2.74 1927.9 2051.8 8.114 308.2 5.88

LTHC 2.74 2022.0 2052.2 7.785 701.1 5.88

HT 7.65 1926.8 2053.9 8.047 373.4 5.88

HTCH 7.65 2017.2 2058.2 7.755 778.2 5.88

MR17 St. 89 LT 1.51 1909.5 2034.3 8.123 295.0 7.54

LTHC 1.51 2005.2 2033.4 7.782 689.7 7.54

HT 4.75 1909.7 2034.8 8.072 340.1 7.54

HTCH 4.75 1999.0 2035.1 7.765 733.3 7.54

MR17 St. 102 LT 6.59 2017.1 2181.3 8.113 325.5 4.21

LTHC 6.59 2117.6 2180.9 7.815 693.4 4.21

HT 10.55 2016.4 2184.2 8.061 377.8 4.21

HTCH 10.55 2112.3 2180.7 7.775 781.4 4.21

OS18 St. 3 LT 7.14 1957.3 2191.8 8.249 226.8 8.93

LTHC 7.14 2083.6 2209.2 7.987 456.4 8.93

HT 11.14 1960.7 2205.3 8.205 258.0 8.93

HTCH 11.14 2074.5 2187.4 7.893 582.8 8.93

OS18 St. 11 LT 8.07 1942.1 2259.5 8.367 167.1 17.14

LTHC 8.07 2052.7 2256.1 8.150 304.2 17.14

HT 12.26 1946.1 2256.0 8.290 206.9 17.14

HTCH 12.26 2040.1 2263.4 8.126 326.5 17.14

OS18 St. 19 LT 10.63 2052.0 2161.0 7.919 545.5 14.27

LTHC 10.63 2152.1 2153.0 7.550 1351.4 14.27

HT 14.74 2047.5 2166.5 7.887 601.3 14.27

HTCH 14.74 2133.3 2169.5 7.621 1170.2 14.27

OS18 St. 30 LT 4.57 2034.9 2208.1 8.145 299.7 20.34

LTHC 4.57 2134.9 2198.0 7.828 667.2 20.34

HT 8.82 2025.8 2218.7 8.124 321.6 20.34

HTCH 8.82 2128.5 2200.0 7.794 743.4 20.34
