# Supplementary material for: Temperature and CO2 alter trophic structure of Arctic plankton assemblages
Source: Sci Rep. 2025 Aug 20;15:28582. doi: 10.1038/s41598-025-10591-0 (PMC12365226; doi:10.1038/s41598-025-10591-0)
Supplement: Supplementary file 2 — Supplementary Material 2 [file 41598_2025_10591_MOESM2_ESM.docx]

Supplemental Table 6. Statistical results of multiple regression analysis using the following equation: *µ*_(large- or small-sized chl-_*_a_*_)_ = a × Temperature + b × pH + c × Temperature × pH + d. Variables which were significant in 2-way ANOVA (Table S1) were included with forced entry method. Numbers in parenthesis represent standardized coefficient. N.S.: not significant.

Exp. Component a (Temp.) b (pH) c (Temp. × pH) Statistics

MR17 St. 4 >10 µm chl-*a* 0.145 (0.95) 0.372 (0.30) N.S. *F*_2,5_ = 253, R^2^ = 0.99, *p* < 0.001

<10 µm chl-*a* 0.097 (0.96) N.S. N.S. *F*_1,6_ = 74.4, R^2^ = 0.91, *p* < 0.001

MR17 St. 21 >10 µm chl-*a* 0.082 (0.76) N.S. N.S. *F*_1,6_ = 8.22, R^2^ = 0.51, *p* = 0.029

<10 µm chl-*a* 0.102 (0.89) −0.307 (−0.39) N.S. *F*_2,5_ = 269, R^2^ = 0.99, *p* < 0.001

MR17 St. 74 >10 µm chl-*a* 0.099 (0.98) N.S. N.S. *F*_1,6_ = 160, R^2^ = 0.96, *p* < 0.001

<10 µm chl-*a* 0.122 (0.97) N.S. N.S. *F*_1,6_ = 110, R^2^ = 0.94, *p* < 0.001

MR17 St. 89 >10 µm chl-*a* 0.142 (0.94) 0.51 (0.34) N.S. *F*_2,5_ = 35.4, R^2^ = 0.91, *p* = 0.001

<10 µm chl-*a* 0.152 (0.75) N.S. N.S. *F*_1,6_ = 7.71, R^2^ = 0.49, *p* = 0.032

MR17 St. 102 >10 µm chl-*a* 0.148 (0.98) N.S. N.S. *F*_1,6_ = 150, R^2^ = 0.96, *p* < 0.001

<10 µm chl-*a* 0.949 (9.7) 0.618 (0.47) −0.108 (−8.7) *F*_3,4_ = 126, R^2^ = 0.98, *p* < 0.001

OS18 St. 3 >10 µm chl-*a* −0.073 (−1.0) N.S. 0.016 (1.9) *F*_2,9_ = 8.94, R^2^ = 0.59, *p* = 0.007

<10 µm chl-*a* 0.102 (0.94) −0.250 (−0.17) N.S. *F*_2,9_ = 352, R^2^ = 0.99, *p* < 0.001

OS18 St. 11 >10 µm chl-*a* −3.37 (−26) −4.70 (−1.7) 0.424 (27) *F*_3,8_ = 206, R^2^ = 0.98, *p* < 0.001

<10 µm chl-*a* 0.095 (0.99) N.S. N.S. *F*_1,10_ = 502, R^2^ = 0.98, *p* < 0.001

OS18 St. 19 >10 µm chl-*a* 0.100 (0.91) 0.488 (0.35) N.S. *F*_2,9_ = 695, R^2^ = 0.99, *p* < 0.001

<10 µm chl-*a* 0.130 (0.99) N.S. N.S. *F*_1,10_ = 685, R^2^ = 0.98, *p* < 0.001

OS18 St. 30 >10 µm chl-*a* 0.126 (0.99) N.S. N.S. *F*_1,10_ = 608, R^2^ = 0.98, *p* < 0.001

<10 µm chl-*a* 0.091 (0.96) 0.402 (0.32) N.S. *F*_2,9_ = 152, R^2^ = 0.97, *p* < 0.001
