# Supplementary material for: Temperature and CO2 alter trophic structure of Arctic plankton assemblages
Source: Sci Rep. 2025 Aug 20;15:28582. doi: 10.1038/s41598-025-10591-0 (PMC12365226; doi:10.1038/s41598-025-10591-0)
Supplement: Supplementary file 3 — Supplementary Material 3 [file 41598_2025_10591_MOESM3_ESM.docx]

Supplemental Table 4. Statistical results of two-way analysis of variance between environmental variables (temperature or *p*CO_2_) and size difference, and the temperature or CO_2_ sensitivity of phytoplankton growth or grazing mortality rates. N.S.: not significant.

Sensitivity Component Size difference pH Size × pH

Temperature phytoplankton N.S. N.S. *p* = 0.002

grazing rate N.S. N.S. N.S.

Size difference Temperature Size × Temperature

CO_2_ phytoplankton N.S. N.S. *p* < 0.001

grazing rate *p* = 0.022 N.S. *p* = 0.025

Supplemental Table 5. Statistical results of two-way analysis of variance between environmental variables (temperature and pH) and the growth rate of phytoplankton on the basis of chlorophyll-*a* concentrations. N.S.: not significant.

Exp. Component Temp. pH Temp. × pH

MR17 St. 4 >10 µm chl-*a* *p* < 0.001 *p* < 0.001 N.S.

<10 µm chl-*a* *p* = 0.002 N.S. N.S.

>10 µm grazing *p* = 0.011 *p* < 0.001 N.S.

<10 µm grazing N.S. N.S. N.S.

MR17 St. 21 >10 µm chl-*a* *p* = 0.026 N.S. N.S.

<10 µm chl-*a* *p* < 0.001 *p* = 0.001 N.S.

>10 µm grazing N.S. N.S. N.S.

<10 µm grazing *p* < 0.001 N.S. N.S.

MR17 St. 74 >10 µm chl-*a* *p* < 0.001 N.S. N.S.

<10 µm chl-*a* *p* < 0.001 N.S. N.S.

>10 µm grazing N.S. N.S. N.S.

<10 µm grazing N.S. N.S. N.S.

MR17 St. 89 >10 µm chl-*a* *p* < 0.001 *p* = 0.013 N.S.

<10 µm chl-*a* *p* = 0.036 N.S. N.S.

>10 µm grazing *p* = 0.002 N.S. N.S.

<10 µm grazing N.S. N.S. N.S.

MR17 St. 102 >10 µm chl-*a* *p* < 0.001 N.S. N.S.

<10 µm chl-*a* *p* < 0.001 *p* = 0.010 *p* = 0.038

>10 µm grazing *p* = 0.047 N.S. N.S.

<10 µm grazing *p* = 0.002 N.S. N.S.

OS18 St. 3 >10 µm chl-*a* *p* = 0.001 N.S. *p* = 0.038

<10 µm chl-*a* *p* < 0.001 *p* = 0.001 N.S.

>10 µm grazing *p* = 0.004 N.S. *p* = 0.027

<10 µm grazing *p* < 0.001 *p* = 0.004 N.S.

OS18 St. 11 >10 µm chl-*a* *p* < 0.001 *p* = 0.002 *p* < 0.001

<10 µm chl-*a* *p* < 0.001 N.S. N.S.

>10 µm grazing *p* < 0.001 *p* = 0.025 *p* = 0.018

<10 µm grazing *p* < 0.001 N.S. N.S.

OS18 St. 19 >10 µm chl-*a* *p* < 0.001 *p* < 0.001 N.S.

<10 µm chl-*a* *p* < 0.001 N.S. N.S.

>10 µm grazing *p* < 0.001 *p* < 0.001 *p* < 0.001

<10 µm grazing *p* < 0.001 *p* = 0.010 *p* = 0.024

OS18 St. 30 >10 µm chl-*a* *p* < 0.001 N.S. N.S.

<10 µm chl-*a* *p* < 0.001 *p* < 0.001 N.S.

Table S5. Continued.

Exp. Component Temp. pH Temp. × pH

OS18 St. 30 >10 µm grazing *p* = 0.034 N.S. N.S.

<10 µm grazing *p* = 0.042 *p* = 0.006 N.S.
