# Supplementary material for: Temperature and CO2 alter trophic structure of Arctic plankton assemblages
Source: Sci Rep. 2025 Aug 20;15:28582. doi: 10.1038/s41598-025-10591-0 (PMC12365226; doi:10.1038/s41598-025-10591-0)
Supplement: Supplementary file 4 — Supplementary Material 4 [file 41598_2025_10591_MOESM4_ESM.docx]

Supplemental Table 1. Environmental conditions at the sampling sites.

Cruise Stn. No. Lat. Long. Time Temp. Salinity DIC TA NO_3_ PO_4_  Si(OH)_4_

(N) (W) (YYYY/mm/dd) (°C) (µmol kg^−1^) (µmol kg^−1^) (µmol kg^−1^) (µmol kg^−1^) (µmol kg^−1^)

MR17 4 65°29’ 169°35’ 2017/08/27 5.10 32.65 2044.7 2240.6 4.56 0.92 9.09

MR17 21 72°48’ 161°21’ 2017/08/30 4.03 31.30 1969.1 2172.8 0.04 0.54 2.22

MR17 74 73°19’ 160°52’ 2017/09/10 2.89 28.05 1927.5 2048.2 0.05 0.48 3.20

MR17 89 75°15’ 177°43’ 2017/09/16 −1.27 29.18 1909.8 2039.8 0.02 0.86 4.12

MR17 102 68°18’ 167°03’ 2017/09/19 6.69 30.71 1988.1 2168.0 0.03 0.59 5.88

OS18 3 62°44’ 174°40’ 2018/07/02 6.85 31.84 2191.8 1957.3 0.36 0.52 1.90

OS18 11 63°54’ 172°14’ 2018/07/04 8.27 32.70 2259.5 1942.1 0.15 0.13 0.20

OS18 19 64°30’ 166°31’ 2018/07/06 10.71 29.53 2161.0 2052.0 0.71 0.56 6.60

OS18 30 66°43’ 168°57’ 2018/07/11 4.82 34.48 2208.1 2034.9 0.80 0.92 5.23

Supplemental Table S2. Biological data at the beginning of the experiments. Abbreviations: LChl-*a* and SChl-*a* represent large- (>10 µm) and small- (<10 – GF/F) chlorophyll-*a*, respectively. rLChl-*a*: the fraction of LChl-*a* to the total chlorophyll-*a*; Syn., PicoE, NanoE and MZ represent *Synechococcus*, pico-eukaryotic phytoplankton, nano-eukaryotic phytoplankton, and microzooplankton, respectively.

Cruise Stn. No. LChl-*a* SChl-*a* rLChl-*a* Syn. PicoE NanoE Diatoms MZ SChl-*a*/MZ ratio

(µg L^−1^) (µg L^−1^) (%) (cells mL^−1^) (cells mL^−1^) (cells mL^−1^) (µmol C L^−1^) (µmol C L^−1^) (g mol^−1^ C)

MR17 4 4.40 0.61 88 3517 1170 2137 241 10.8 0.06

MR17 21 0.17 0.12 59 347 2030 567 2.17 0.76 0.15

MR17 74 0.37 0.24 61 497 6633 503 0.88 1.51 0.16

MR17 89 0.18 0.05 78 87 180 90 2.18 0.63 0.08

MR17 102 0.77 1.04 42 84767 25953 3900 6.45 1.30 0.80

OS18 3 0.15 0.24 39 790 8640 3200 2.40 2.65 0.09

OS18 11 0.04 0.23 14 425 6955 1030 1.45 1.80 0.13

OS18 19 0.99 0.96 51 1330 14330 1780 98.3 5.59 0.20

OS18 30 1.24 0.47 72 1060 4145 2380 4.29 1.80 0.31
