# Supplementary material for: Temperature and CO2 alter trophic structure of Arctic plankton assemblages
Source: Sci Rep. 2025 Aug 20;15:28582. doi: 10.1038/s41598-025-10591-0 (PMC12365226; doi:10.1038/s41598-025-10591-0)
Supplement: Supplementary file 6 — Supplementary Material 6 [file 41598_2025_10591_MOESM6_ESM.docx]

Supplemental figure and table captions on Temperature and CO_2_ alter trophic structure of Arctic plankton assemblages

Koji Sugie, Bingzhang Chen, Shigeto Nishino, Toru Hirawake

**Captions**

Supplemental fig. 1. Map of sampling stations in the western Arctic region.

Supplemental fig. 2. Graphical illustration of the method showing the four series of the two-point dilution technique. Abbreviations: SW: seawater; FSW: filtered seawater; DIC: dissolved inorganic carbon: LT: lower temperature control; LTHC: lower temperature plus high CO_2_ treatment; HT: LT plus 4°C treatment; HTHC: LTHC plus 4°C treatment.

Supplemental fig. 3. Specific growth rate of >10 µm phytoplankton (a–e) and their grazing mortality rate (f–j) at each sampling station in the 2017 experiments. White and gray bars represent lower and higher temperature treatments, respectively. Open and slashed bars represent CO_2_ unamended controls and high CO_2_ treatments, respectively. Error bars represent 1SD of duplicate (2017-experiment) or triplicate (2018-experiment) bottles. The letters above the bars represent the statistical results of two-way ANOVA and post-hoc multiple comparisons with the Holm-Bonferroni method.

Supplemental fig. 4. Similar to the Supplemental fig. 3 but for smaller phytoplankton and grazing mortality rates at each sampling station in the 2017 experiment.

Supplemental fig. 5. Similar to the Supplemental fig. 3 but for the 2018 experiments.

Supplemental fig. 6. Similar to the Supplemental fig. 4 but for the 2018 experiments.

Supplemental fig. 7. Scatter plot of *in situ* *p*CO_2_ of the initial seawater sample and CO_2_ response index of the large phytoplankton traits. Solid and thin lines represent significant regressions and their 95% CL. CO_2_ response in control temperature = −0.011 + 44.7e^−0.029•^*^p^*^CO2^ (*p* < 0.001, *F*_2,19_ = 99.5, R^2^ = 0.90); and that in higher temperature = 4.0 × 10^−4^ × *p*CO_2_ – 0.14 (*p* = 0.002, *F*_1,17_ = 12.7, R^2^ = 0.39: excluded highest *p*CO_2_ data).

Supplemental fig. 8. Relationships between the growth rate of larger phytoplankton and their grazing mortality rate at each station in the 2017 (left panels) and 2018 (right panels) experiments.

Supplemental fig. 9. Similar to the Supplemental fig. 8 but for smaller phytoplankton traits.

Supplemental fig. 10. The relationship among phytoplankton growth rate (µ) at reference temperature, temperature response as estimated from linearity as conducted in the present study, and Q_10_. Given that the linearity constant is ~0.1 to 0.13 as observed in the present study, the Q_10_ value can far exceed the canonical value of 1.88 when the growth rate of phytoplankton is low such as in the low-temperature polar region.
